# The role of machine learning algorithms in detection of gestational diabetes; a narrative review of current evidence

**DOI:** 10.1186/s40842-024-00176-7

**Published:** 2024-06-25

**Authors:** Emmanuel Kokori, Gbolahan Olatunji, Nicholas Aderinto, Ifeanyichukwu Muogbo, Ikponmwosa Jude Ogieuhi, David Isarinade, Bonaventure Ukoaka, Ayodeji Akinmeji, Irene Ajayi, Ezenwoba Chidiogo, Owolabi Samuel, Habeebat Nurudeen-Busari, Abdulbasit Opeyemi Muili, David B. Olawade

**Affiliations:** 1 Department of Medicine and Surgery, University of Ilorin, Ilorin, PMB 5000 Nigeria; 2 Department of Medicine, Ladoke Akintola University of Technology, Ogbomoso, Nigeria; 3 Department of Medicine, Siberian State Medical University, Tomsk, Russia; 4 Department of Internal Medicine, Asokoro District Hospital, Abuja, Nigeria; 5 Department of Medicine and Surgery, Olabisi Onabanjo University, Ogun, Nigeria; 6 Department of Medicine and Surgery, AfeBabalola University, Ado-Ekiti, Nigeria; 7 Department of Medicine, Lagos State Health Service Commission, Lagos, Nigeria; 8 Department of Allied and Public Health, School of Health, Sport and Bioscience, University of East London, London, UK

**Keywords:** Gestational diabetes mellitus (GDM), Machine learning, Early prediction, Artificial intelligence, Obstetrics

## Abstract

Gestational Diabetes Mellitus (GDM) poses significant health risks to mothers and infants. Early prediction and effective management are crucial to improving outcomes. Machine learning techniques have emerged as powerful tools for GDM prediction. This review compiles and analyses the available studies to highlight key findings and trends in the application of machine learning for GDM prediction. A comprehensive search of relevant studies published between 2000 and September 2023 was conducted. Fourteen studies were selected based on their focus on machine learning for GDM prediction. These studies were subjected to rigorous analysis to identify common themes and trends. The review revealed several key themes. Models capable of predicting GDM risk during the early stages of pregnancy were identified from the studies reviewed. Several studies underscored the necessity of tailoring predictive models to specific populations and demographic groups. These findings highlighted the limitations of uniform guidelines for diverse populations. Moreover, studies emphasised the value of integrating clinical data into GDM prediction models. This integration improved the treatment and care delivery for individuals diagnosed with GDM. While different machine learning models showed promise, selecting and weighing variables remains complex. The reviewed studies offer valuable insights into the complexities and potential solutions in GDM prediction using machine learning. The pursuit of accurate, early prediction models, the consideration of diverse populations, clinical data, and emerging data sources underscore the commitment of researchers to improve healthcare outcomes for pregnant individuals at risk of GDM.

## Introduction

Gestational diabetes mellitus (GDM) is characterised by any degree of glucose intolerance that either develops or is first identified during pregnancy [1]. It encompasses cases of previously undiagnosed glucose intolerance that may have existed before or emerged during pregnancy, regardless of subsequent management approaches, such as dietary modification or insulin therapy, and whether the condition persists post-pregnancy [2]. Regional disparities in GDM prevalence are evident, with the highest rates found in the Middle East and North Africa (12.9%), followed by Southeast Asia (11.7%), the Western Pacific (11.7%), South and Central America (11.2%), and the lowest rates in Europe (5.8%), North America, and the Caribbean (7.0%) [3]. GDM is a widespread pregnancy complication, affecting 1–14% of pregnancies worldwide, with variations influenced by patient ethnicity and diagnostic criteria [4, 5]. The impact of GDM on maternal and fetal health is significant, often leading to preterm delivery, cesarean section, excessive fetal growth, hyperinsulinemia, hypoglycemia, and hyperbilirubinemia in newborns [6–8]. Additionally, GDM can progress to Type 2 Diabetes Mellitus (T2DM), resulting in birth-related complications, visceromegaly, fetal macrosomia, and an increased risk of metabolic disorders for both mother and child, including hypertension, obesity, and metabolic syndrome [9, 10].

The precise pathophysiological mechanisms of GDM remain incompletely understood, but hormonal imbalances, impaired insulin sensitivity, and pancreatic β-cell malfunction are suggested contributors [11]. About 16% of pregnancies globally are linked to hyperglycemia, with 84% classified as GDM [12]. GDM significantly contributes to the onset of T2DM in both mothers and offspring, emphasising the importance of effectively managing blood glucose levels during pregnancy to prevent and reduce the prevalence of T2D in future generations [13]. Historically, screening for GDM relied on medical history, previous obstetric outcomes, and family history of T2D. However, this approach exhibited an approximate 50% failure rate in detecting GDM among pregnant women. In 1973, a pivotal study recommended adopting the 50 g 1-h oral glucose tolerance test as a screening tool, which is now widely used by approximately 95% of obstetricians in the United States for GDM screening. In 2014, the U.S. Preventive Services Task Force (USPSTF) recommended GDM screening for all pregnant women at 24 weeks [12, 14, 15].

Early screening and diagnosis of GDM are crucial for reducing the risks of pregnancy-related complications, such as macrosomia, preterm birth, pre-eclampsia, and neonatal intensive care admissions [14, 16]. Existing diagnostic tools have limitations in this regard. To enhance the prediction of GDM, clinical, sociodemographic, and anthropometric data have been employed in traditional regression analysis-based clinical risk prediction models. Recent advancements in machine learning promise to increase the accuracy of disease perception, diagnosis, and management. For instance, Belsti et al. [17] used a predictive analysis on antenatal care records. Their model achieved 85% accuracy, 90% precision, 78% recall, 84% F1-score, 81% sensitivity, 90% specificity, 92% positive predictive value, 78% negative predictive value, and a Brier Score of 0.39, surpassing the performance of traditional statistical methods. Most outcome prediction models enable early intervention in high-risk women and cost-effective screening by identifying low-risk individuals, potentially eliminating the need for glucose tolerance tests [18]. This review explores the effectiveness of machine learning algorithms in detecting GDM, incorporating relevant studies and data on their application for GDM detection.

## Methodology

### Literature search strategy

A literature search was carried out to review the role of machine learning algorithms in the early detection of GDM and their impact on fetomaternal outcomes. The following databases were searched: PubMed, Scopus, Web of Science, and Google Scholar. The search was conducted for studies published between 2000 and September 2023. The following keywords were used (“machine learning”[MeSH Terms] OR (“machine”[All Fields] AND “learning”[All Fields]) OR “machine learning”[All Fields]) AND (“algorithms”[MeSH Terms] OR “algorithms”[All Fields]) AND (“diabetes, gestational”[MeSH Terms] OR (“diabetes”[All Fields] AND “gestational”[All Fields]) OR “gestational diabetes”[All Fields] OR (“gestational”[All Fields] AND “diabetes”[All Fields] AND “mellitus”[All Fields]) OR “gestational diabetes mellitus”[All Fields]).

### Inclusion and exclusion criteria

Articles were included if they met the following criteria:

- Published in English.
- Peer-reviewed original studies.
- Focused on applying machine learning algorithms in the context of GDM.
- Included information on using machine learning in detecting or predicting GDM.

The exclusion criteria were:

- Systematic analyses, meta-analyses, reviews, conference abstracts, case reports, editorials, and letters.
- Studies that did not provide relevant information or data on the topic.

### Study selection

Two independent reviewers (NA & EK) initially screened titles and abstracts to identify potentially relevant articles. Full-text articles were then retrieved for further evaluation. Discrepancies were resolved through discussion, and a third reviewer (GO) was consulted when necessary.

### Data extraction

Data were extracted from the selected articles, including study design, sample size, characteristics of the study population, machine learning algorithms employed, predictive variables used, outcomes measured, and reported results.

### Data synthesis

The findings from the selected studies were synthesised to provide an overview of the current evidence regarding the role of machine learning algorithms in the early detection of GDM and their impact on fetomaternal outcomes. Common themes, trends, and methodological differences were identified. Results were analysed and presented in a clear and organised manner.

## Results

The studies in this review focused on predicting and detecting GDM through machine learning algorithms (See Table 1). Most were retrospective studies; others were cohort studies, and two were randomised clinical trials. The populations studied vary in size, from smaller cohorts of just a few thousand individuals to larger populations exceeding 30,000. The studies reviewed utilised diverse machine learning algorithms, including Naïve Bayes, Decision Trees, Support Vector Machines, Neural Networks, Logistic Regression, Lasso-Logistics, Gradient Boosting Decision Tree (GBDT), Deep Neural Network (DNN), Gaussian Naïve Bayes (GNB), Bernoulli Naïve Bayes (BNB), and various ensemble methods such as Light Gradient Boosting Machine (LGBM) and Extreme Gradient Boosting (XGBoost). Data sources include pregnancy registries, perinatal databases, clinical records, and data from health institutions or hospitals.

**Table 1:** Characteristics of reviewed studies

**Authors & Year**	**Study Design**	**Objective/Purpose**	**Population & Size**	**Machine learning algorithm**	**Data Sources**	**Outcome Measured**	**Key Findings**
Gabriel Cubillos et al. (2023) [19]	Retrospective study	To develop machine learning (ML) models, for the early prediction of GDM using widely available variables, facilitating early intervention	Dataset used included registries from 1,611 pregnancy	Gaussian Naïve Bayes (GNB) and Bernoulli Naïve Bayes (BNB), Decision Trees (DT), Support Vector Machines (SVMs), Multi-Layer Perceptron (MLP), K-Nearest Neighbors (KNN), Logistic Regression (LR), Random Forest (RF), Extra Trees (ET), Balanced Random Forest (BRF), Gradient Boosting (GB), implemented in Extreme Gradient Boosting (XGB), and Light Gradient Boosting Machine (LGBM)	Pregnancy registry of patients attending the Obstetrics and Fetal Medicine Unit of the Hospital Parroquial de San Bernardo, Santiago, Chile between 2019–2022	Maternal weight, BMI, Age, 1TFG, Chronic hypertension, Gravidity, Parity, Insulin resistance, hypothyroidism, vaginal deliveries, abortion.	Early prediction of GDM within early stages of pregnancy using regular examinations; the development and optimization of twelve different ML models and their hyperparameters to achieve the highest prediction performance; a novel data augmentation method is proposed to allow reaching excellent GDM prediction results with various models.
Jesús et al. 2023 [20]	Prospective Cohort study	To develop an AI-based prediction model for risk of developing GDM among pregnant women in Mexico.	860 women (430 with GDM, and 430 without GDM)	Medición Integrada para la Detección Oportuna (MIDO) AI model for predicting gestational diabetes (MIDO GDM) - Multiple Artificial Neuronal Network (ANN) Algorithms	The *Cuido Mi Embarazo* (CME) study that collected data from 1709 pregnant women in Mexico between April 2019 and May 2021.	Age, Pregestational BMI, Parity, Family history of diabetes mellitus, Family history of hypertension, History of hypertension, Gestational week, Enrolment BMI, Random capillary glucose at study enrollment, and fasting plasma glucose measured between the 24th and 28th week of pregnancy (first OGTT measurement)	The artificial neural network used to build this model achieved a high level of accuracy (70.3%) and sensitivity (83.3%) for identifying women at high risk of developing GDM. This AI-based model was set to be applied throughout Mexico to improve the timing and quality of GDM interventions.
Kang BS et al. (2023) [21]	Retrospective cohort study	To compare the performances of light gradient boosting machine (LGBM) and extreme gradient boosting (XGBoost) algorithms, with a full set of variables in predicting gestational diabetes mellitus (GDM)	34,387 (nulliparous - multiparous women	Gradient boosting machine algorithms (LGBM & XGBoost)	Perinatal database for women who delivered between January 2009 and December 2020 at 7 hospitals in four areas of South Korea	Performances of LGBM and XGBoost across the whole data set, nulliparity, and multiparity cohorts, at four different stages (baseline, E0, E1, and M1)	GDM was diagnosed in 3,103 pregnancies (9.02%) in the entire cohort. XGBoost outperformed LGBM in most cohorts and at most time points, except for the E1
Yi-xin Li et al. (2023) [22]	Cohort study	To use machine learning (ML) algorithms to study data gathered throughout the first trimester in order to predict GDM.	4799 and 2795 women in their first trimester	Extreme gradient boosting (XGBoost)	Pregnant women for the Xinhua Hospital Chongming branch (XHCM) and the Shanghai Pudong New Area People’s Hospital (SPNPH) formed the independent cohorts	Pre-pregnancy BMI and maternal abdominal circumference at pregnancy initiation, and FPG and HbA1c at the end of the first trimester	The model predicted GDM with moderate performance at pregnancy initiation and good-to-excellent performance at the end of the first trimester in the XHCM cohort. The trained XGBoost showed moderate performance in the SPNPH cohort
Jenny Yang et al. (2022) [23]	Retrospective cohort study	To introduce a machine learning-based stratification system for identifying patients at risk of exhibiting high blood glucose levels	1148 pregnant women with GDM at Oxford University Hospital and 709 from Royak Berkshire Hospital	Linear and non-linear tree-based regression models including XGBoost MSE, R2, MAE	Pregnant women with GDM, managed at the OUH, and subscribed to the GDm-Health system between 30 April 2018 to 4 May 2021 Also, GDm-Health data of 709 pregnancy cases at the Royal Berkshire Hospital	4–6 times daily blood sugar check, BMI.	Study outlined and demonstrated a straightforward method for implementing proportionate care delivery based on features already existing in GDM clinics
Jie Zhang et al. (2022) [24]		To predict Gestational DM under Cascade and Ensemble Learning Algorithm	1000 training samples and 85-dimensional features	Logistics regression model, Lasso-Logistics, Gradient Boosting Decision Tree (GBDT), Extreme Gradient Boosting (Xgboost), Light Gradient Boosting Machine (Lightgbm), and Gradient Boosting Categorical Features (Catboost)	Data set commissioned by Beijing Qingwutong Health Technology Company published in the Tianchi Big Data Competition held by Alibaba	Physical indicators, such as age, height, weight, BMI, and cholesterol indicators. The other 55 are genetic features	Data set utilized in this work, the accuracy of the proposed prediction model is 80.3%, the precision is 74.6%, and the recall rate is 79.3%.
Lauren D Liao et al. (2022) [25]	Population-based cohort study	To investigate whether clinical data at varied stages of pregnancy can predict GDM treatment modality. To predict risks for pharmacologic treatment beyond MNT(medical nutrition therapy)	30,474 pregnant women with GDM	Transparent and Ensemble machine learning prediction methods, including LASSO regression and super learner, containing classification and regression tree, LASSO regression, random forest, and extreme gradient boosting algorithms	Pregnant women with GDM delivered at Kaiser Permanente Northern California between 2007–2017 (KPNC Pregnancy Glucose Tolerance and GDM Registry)	Responsiveness to MNT, then to OHA and with insulin	Clinical data demonstrated reasonably high predictability for GDM treatment modality at the time of GDM diagnosis and high predictability at 1-week post GDM diagnosis
Mukkesh Kumar et al. (2022) [26]	Cohort study.	To evaluate the predictive ability of existing UK NICE guidelines for assessing GDM using machine learning	909 pregnant women	CatBoost gradient boosting algorithm, and the Shapley feature attribution framework	GUSTO (Growing Up in Singapore Towards healthy Outcomes) prospective multi-ethnic mother–offspring pregnant women recruited at 7–11 weeks of gestational age.	Mean arterial blood pressure in *first trimester*, age, ethnicity and previous history of GDM	UK NICE guidelines were insufficient to assess GDM risk in Asian women. The non-invasive predictive model developed in this study outperformed the current state-of-the-art machine learning models to predict GDM
Mukkesh Kumar et al. (2022) [27]	Prospective (preconception) cohort study.	To build a preconception-based GDM predictor to enable early intervention. To also assess the associations of top predictors with GDM and adverse birth outcomes	1032 Women planning for pregnancies were recruited from the KK Women’s and Children’s Hospital (KKH) and community of multi-ethnic groups (Chinese, Malay, Indian or any combination of these three ethnicities)between February 2015 and October 2017	Evolutionary algorithm-based automated machine learning (AutoML) - SHAP framework + TPOT	Mother–child dyads were followed for 7 years, with longitudinal phenotypic data collected across multiple health domains.	Demographics, medical/obstetric history, physical measures, blood-derived markers, lifestyle factors and antenatal OGTT	The study devised a population-based predictive care solution to assess the risk of developing GDM in preconception of Asian women
Yuhan Du et al. (2022) [28]	Randomized Clinical Trial	To apply machine learning to develop a clinical decision support system (CDSS) that predicts the risk of GDM in a high risk group of women with overweight and obesity	1,139 pregnant women (186 with GDM) from eastern China	Random Forest model and Logistic Regression model	The study was conducted at three primary women and child health care centres and a university-affiliated hospital.	Pre-pregnancy BMI, abdomen circumference in the first trimester, age, PCOS, gravidity,	The research developed a simple model to predict the risk of GDM using machine learning algorithm in the first trimester without blood examination indexes
Li-Li Wei et al. (2021) [29]	Retrospective study	To study the application of a machine learning algorithm for predicting gestational diabetes mellitus (GDM) in early pregnancy	1625 Pregnant women who had attended medical institutions for an antenatal examination in pregnant women in the Qingdao area of China from November 2017 to August 2018	Random Forest regression algorithm	Face-to-face questionnaire survey of participants and review of pregnancy-related medical records to obtain indicators related to GDM	BMI, Pregnancy weight, Blood group, Blood pressure, comorbidities	The variables of body weight at birth and mother’s weight were identified to be strongly predictive of GDM in all models. Other variables (e.g., colpomycosis, kidney disease, number of births by the mother, regular menstruation, blood type, and hepatitis) that consistently ranked in the top 20 most influential factors were also found to be linked to GDM in this study
Yang-Ting Wu et al. (2021) [30]	Retrospective study	To establish effective models to predict early GDM.	16 819 cases in the training data set, and 15 371 cases in the testing data set	Logistic regression (LR), K-nearest neighbor (KNN), Support vector machine (SVM), and Deep neural network (DNN)	2017 Obstetrical electronic medical record data from the International Peace Maternal and Child Health Hospital, Shanghai Jiao Tong University School of Medicine	Advanced maternal age, body mass index (BMI), and family history of diabetes, Blood pressure, Parity	A clinically cost-effective 7-variable LR model was developed. The relationship of GDM with thyroxine and BMI was also investigated in the Chinese population.
de Freitas et al. 2020 [31]	Case–control study	To investigate the use of attenuated total reflection Fourier-transform infrared (ATR-FTIR) spectroscopy to analyse spectrochemical information using chemometric methods for accurate and low-cost GDM detection.	50 GDM women with single pregnancies at a gestational age of between 12 and 38 weeks and 50 healthy pregnant control group at a Reference Obstetrics and Gynecology Hospital between January and October 2018.	Chemometric approaches, including feature selection algorithms associated with discriminant analysis, such as Linear Discriminant Analysis (LDA), Quadratic Discriminant Analysis (QDA) and Support Vector Machines (SVM)	Pregnant women at a Reference Obstetrics and Gynecology Hospital between January and October 2018.	Age, BMI by classification (suitable, low weight, overweight, obesity), marital status and parity, Previous mode of delivery, GDM history, family history of GDM, History of disease in pregnancy	The Fourier-transform infrared (FTIR) spectra of blood plasma samples taken from pregnant women with GDM can rapidly distinguish diabetic cohorts from healthy pregnant women. Using the Genetic Algorithm Linear Discriminant Analysis (GA-LDA) method, GDM could be distinguished from healthy pregnant controls with 100% accuracy, sensitivity, and specificity in an external test set.
Yunzhen Ye et al. (2020) [32]	Retrospective Cohort Study	To use machine learning methods to predict GDM and compare their performance with that of logistic regressions.	22,242 singleton pregnancies were included, and 3182 (14.31%) women developed GDM	GDBT, AdaBoost, LGB, Logistic, Vote, XGB, Decision Tree, and Random Forest	Obstetrics and Gynecology Hospital of Fudan University in China from 2013 to 2017.	Primary outcome was GDM. Secondary outcomes included adverse pregnancy outcomes, including cesarean delivery for any reason, preeclampsia, macrosomia, IUGR, preterm birth (≤ 34 gestational weeks), neonatal asphyxia, and perinatal death.	This study found that several machine learning methods did not outperform logistic regression in predicting GDM. A model with cutoff points for risk stratification of GDM was also developed.
Jingyuan Wang et al. 2021 [33]	Prospective cohort study	To develop and verify an early prediction model of gestational diabetes mellitus (GDM) using machine learning algorithm	2811 pregnant women in eastern China, from 2017 to 2019	Logical Regression (LR), Random Forest (RT), Articial Neural Network (ANN) and Support Vector Machine (SVM)	Dataset was derived from a cohort of pregnant women in Qingdao between November 2017 and December 2019	Socio-demographic characteristics and medical history, including age (identied from the identity card), height, pre-pregnancy body weight, and family history of diabetes. gravidity, parity, multiple birth (yes/no), and pregnancy complications), as well as laboratory test results, including Hemoglobin (Hb), Urine Ket (U-Ket), Fasting Plasma Glucose (FPG), triglyceride (TG), total cholesterol (TC), and HighDensity Lipoprotein (HDL)	Study constructed a New-Stacking model theoretically, for its best performance in specificity, accuracy and AUC. But the SVM model achieved the best performance in sensitivity

### Model performance and comparison

The studies conducted by Kang et al. (2023) and Yunzhen et al. (2020) demonstrated notable outcomes in terms of model performance and comparison [21, 32]. Kang et al. [21] conducted an analysis comparing the effectiveness of two machine learning algorithms, namely Light Gradient Boosting Machine (LGBM) and XGBoost, in predicting GDM. This study revealed that XGBoost consistently outperformed LGBM when evaluated across diverse cohorts and time points, positioning it as a promising choice for the prediction of GDM. In contrast, Yunzhen et al. [32] explored the potential of machine learning methods to surpass traditional logistic regression in GDM prediction. Their results, however, indicated that several machine-learning methods fell short of outperforming logistic regression.

Jie et al. [24] implemented diverse machine learning algorithms, including Logistic Regression, Gradient Boosting Decision Tree, XGBoost, and Lightgbm. The outcome was a model with high accuracy, precision, and recall, demonstrating the potential of these algorithms to enhance GDM prediction and risk assessment. Complementing this, Yang-Ting et al. [30] introduced a clinically cost-effective 7-variable Logistic Regression model. This simplified approach offers a promising avenue for GDM prediction, making it accessible and practical for clinical applications.

### Early prediction

Gabriel et al. [19] develop employed Gaussian Naïve Bayes (GNB), Bernoulli Naïve Bayes (BNB), Decision Trees (DT), Support Vector Machines (SVMs), Multi-Layer Perceptron (MLP) to predict early prediction of GDM within the early stages of pregnancy through regular examinations. The results showed that the developed ML models and the proposed data augmentation method achieved excellent predictive performance for GDM. Similarly, Jenny et al. [23] introduced a novel machine learning-based stratification system. The study utilised linear and non-linear tree-based regression models, including XGBoost. The study demonstrated a straightforward method for implementing proportionate care delivery based on existing features in GDM clinics. The machine learning-based stratification system identified patients at risk of high blood glucose levels, enhancing the ability to tailor care interventions. Furthermore, Yi-xin et al. [22] utilised machine learning to forecast GDM risk with a moderate performance at pregnancy initiation, ultimately achieving good-to-excellent predictive capabilities by the end of the first trimester. The ML algorithm utilised in the study was XGBoost. The machine learning model demonstrated moderate performance in predicting GDM at pregnancy initiation and good-to-excellent performance at the first cohort’s end of the first trimester. However, in the second cohort, the trained XGBoost exhibited moderate performance. The primary objective of the prospective cohort study conducted by Jingyuan Wang et al. [33] was to develop and verify an early prediction model for GDM using machine learning algorithms. Various machine learning algorithms, including LR, Random Forest (RT), ANN, and SVM, were employed in the study. The study findings indicate that the constructed New-Stacking model theoretically aimed for optimal specificity, accuracy, and AUC. Nonetheless, the SVM model demonstrated superior performance, specifically in sensitivity.

Jesús et al. [20] conducted a study to address the barriers to early detection of GDM in pregnant Mexican women. The study employed a machine-learning-driven method to select the best predictive variables for GDM risk. The identified variables included age, family history of type 2 diabetes, previous diagnosis of hypertension, pregestational body mass index, gestational week, parity, birth weight of the last child, and random capillary glucose.

Subsequently, an artificial neural network approach was used to build the AI-based prediction model. The developed model demonstrated a high level of accuracy, reaching 70.3%, and sensitivity, achieving 83.3%. These results indicate the model’s effectiveness in identifying pregnant women at high risk of developing GDM. Moreover, de Freitas et al. [31] conducted a study aiming to characterise GDM in pregnant women better using Attenuated Total Reflection Fourier-transform infrared (ATR-FTIR) spectroscopy. The study employed chemometric approaches, integrating feature selection algorithms along with discriminant analysis methods such as Linear Discriminant Analysis (LDA), Quadratic Discriminant Analysis (QDA), and Support Vector Machines (SVM). The results obtained by Genetic Algorithm Linear Discriminant Analysis (GA-LDA) were reported as the most satisfactory, achieving % accuracy, sensitivity, and specificity of 100%.

### Results in diverse populations

Mukkesh Kumar et al. [26] conducted a cohort study to evaluate the predictive ability of the existing UK National Institute for Health and Care Excellence (NICE) guidelines for assessing GDM using machine learning. This study employed the CatBoost gradient boosting algorithm and the Shapley feature attribution framework for predictive modelling. The findings of the study revealed that the existing UK NICE guidelines were insufficient to assess GDM risk in Asian women. Furthermore, the non-invasive predictive model developed in this study demonstrated superior performance to the current state-of-the-art machine learning models in predicting GDM. Similarly, Mukkesh Kumar et al. [27] built a preconception-based GDM predictor to enable early intervention. Additionally, the study aimed to assess the associations of top predictors with GDM and adverse birth outcomes. Participants were recruited from multi-ethnic groups (Chinese, Malay, Indian, or any combination of these three ethnicities). The study employed an evolutionary algorithm-based automated machine learning (AutoML) approach, incorporating the SHAP (SHapley Additive exPlanations) framework and TPOT (Tree-based Pipeline Optimization Tool). The study successfully devised a population-based predictive care solution, utilising an AutoML approach, to assess the risk of developing GDM among Asian women in the preconception period. While effective in some contexts, their findings revealed that these algorithms proved insufficient for accurately assessing GDM risk in some ethnic groups of women. This study highlights the need for population-specific considerations when addressing GDM.

### Predictive models for specific cohorts

Yuhan et al. [28] conducted a Randomized Clinical Trial to apply machine learning techniques to develop a Clinical Decision Support System (CDSS). The objective was to predict the risk of Gestational Diabetes Mellitus (GDM), specifically in a high-risk group of women with overweight and obesity.. The study employed both Random Forest and Logistic Regression models for prediction. The study successfully developed a simple yet effective model utilising machine learning algorithms to predict the risk of GDM in the first trimester. Notably, the model achieved this without relying on blood examination indexes. Li-Li et al. [29] conducted a retrospective study to investigate the application of a machine learning algorithm for predicting GDM in early pregnancy. The machine learning algorithm employed in the study was the Random Forest regression algorithm. Notably, the model identified body weight at birth and the mother’s weight as strongly predictive variables for GDM. Additionally, other variables such as colpomycosis, kidney disease, the number of births by the mother, regular menstruation, blood type, and hepatitis consistently ranked among the top 20 most influential factors. They were found to be linked to GDM in the study.

### Clinical data and treatment modality

Lauren et al. [25] conducted a population-based cohort study to investigate whether clinical data at different stages of pregnancy could predict the treatment modality for GDM. The focus of the study was on predicting the risks for pharmacologic treatment beyond medical nutrition therapy (MNT) for pregnant women diagnosed with GDM. The study employed transparent and ensemble machine learning methods for predictive modelling, incorporating LASSO regression and a super learner. The super learner included classification, regression tree, LASSO regression, random forest, and extreme gradient boosting algorithms. The study’s findings demonstrated reasonably high predictability for GDM treatment modality at GDM diagnosis and maintained high predictability at 1-week post-GDM diagnosis. In parallel, Jenny et al. [23] demonstrated the development of an innovative method for implementing proportionate care delivery based on existing features within GDM clinics. For predictive modelling, the study employed linear and non-linear tree-based regression models, including metrics such as XGBoost MSE (Mean Squared Error), R2 (R-squared), and MAE (Mean Absolute Error). The findings suggest that such a machine learning-based stratification system could provide an effective and practical approach for tailoring care interventions based on existing features within GDM clinics, potentially improving patient outcomes and resource allocation.

## Discussion

The studies reviewed here encompass various methodologies, underlining the multifaceted nature of GDM prediction. One striking trend within this collection of studies is the detailed comparison of machine learning algorithms. Algorithms like XGBoost and Logistic Regression have demonstrated their effectiveness in GDM prediction [29]. However, it is essential to recognise that there is no one-size-fits-all solution. While XGBoost displayed superiority in several studies, comprehending the strengths and weaknesses of different algorithms becomes crucial for optimising predictive models within various contexts.

**Fig. 1:**
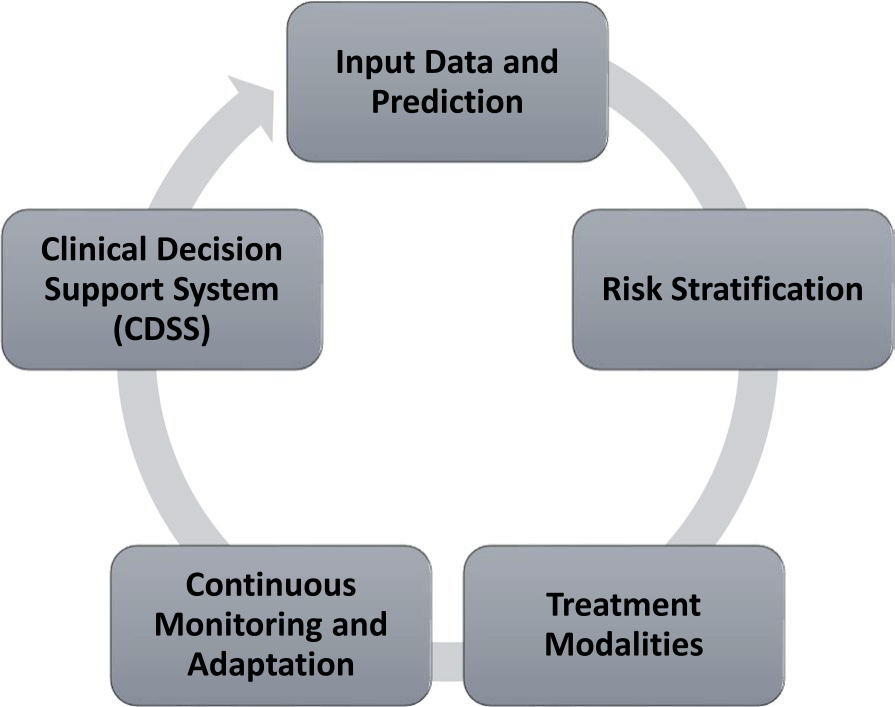
Translating machine learning predictions into clinical interventions for gestational diabetes

The importance of early prediction for effective GDM management cannot be overstated, and it is evident in the significant emphasis placed on this aspect in the reviewed studies [25, 34] (Fig. 1). The rationale behind early prediction lies in the potential to initiate timely interventions and provide personalised care to pregnant women at risk of developing GDM. The complications associated with GDM can have profound and long-lasting effects on both the mother and child, making early detection a critical component of effective healthcare [35]. This emphasis on early prediction is reflected in the proliferation of diverse models designed to forecast GDM risk during the early stages of pregnancy. The variety of models exemplified by the comprehensive work of Gabriel Cubillos et al. [19] underscores the collective ambition within the scientific community to enhance the accuracy and reliability of GDM predictions. The study by Gabriel Cubillos and their team is particularly noteworthy as it prioritised early prediction and explored the potential of different machine-learning models [19]. They expanded the toolkit for healthcare providers and researchers by developing and optimising twelve distinct models. These models are fine-tuned to deliver high prediction performance during the early stages of pregnancy. This multi-pronged approach allows for more comprehensive risk assessment, increasing the chances of timely interventions. The focus on early prediction is not only about identifying cases but also about developing a deeper understanding of the factors and variables that contribute to the development of GDM [36]. By emphasising the importance of early detection, these studies pave the way for tailoring interventions that can prevent or mitigate the impact of GDM. The ultimate goal is to improve maternal and fetal health outcomes by making proactive, personalised care a standard practice in obstetrics.

Studies within this review underscore the importance of tailoring predictive models to specific populations and demographic groups when addressing the prediction and early detection of GDM [19, 23, 30]. These studies highlight that a one-size-fits-all approach is insufficient, and demographic-specific considerations are essential for constructing accurate predictive models. Mukkesh Kumar et al. [26] have made a particularly striking contribution by shedding light on the limitations of employing uniform guidelines for diverse populations, specifically emphasising the challenges faced by Asian women. Their findings reveal that traditional, broadly applicable guidelines may not adequately capture the unique risk factors and nuances associated with GDM in Asian populations. This study emphasises the necessity of considering ethnicity, genetics, and other demographic-specific factors when constructing predictive models for GDM. By doing so, healthcare providers can better identify at-risk individuals within these populations and tailor interventions and care strategies to their specific needs. Similarly, the research conducted by Yuhan Du et al. (2022) provides a compelling illustration of the potential for augmenting prediction accuracy by focusing on high-risk groups [23]. In this case, the study zeroes in on women who are overweight or obese, a demographic with a higher susceptibility to GDM. By developing a specialised clinical decision support system for this specific cohort, the study recognises the unique risk profile of these individuals. This targeted approach can enhance prediction accuracy, ensuring women at the highest risk receive the necessary attention, interventions, and care. These findings indicate the importance of healthcare equity, emphasising that predictive models must be sensitive to the diversity of the populations they serve. The one-size-fits-all approach is no longer adequate, as demographic factors significantly determine GDM risk. Future research and healthcare initiatives should consider these demographic-specific considerations when designing predictive models, ultimately leading to more accurate risk assessment and better-tailored interventions.

Lauren et al. (2022) and Jenny et al. (2022) made substantial contributions to the field by emphasising the importance of integrating clinical data into the predictive models for GDM [23, 25]. These studies provide valuable insights into how leveraging clinical data can enhance the treatment and care delivery for individuals diagnosed with GDM, ultimately improving patient outcomes. The integration of clinical data into predictive models offers several crucial advantages. First and foremost, it enables healthcare providers to personalise and optimise the treatment and care for pregnant individuals diagnosed with GDM. By considering clinical data such as responsiveness to medical nutrition therapy, they can tailor interventions to each patient’s specific needs. This individualised approach is essential, as GDM management can vary significantly from one person to another [37]. Furthermore, incorporating clinical data fosters a more patient-centred approach to care. It ensures that the treatment plan aligns with the patient’s specific health profile, preferences, and response to interventions. This patient-centred approach can improve patient satisfaction, compliance, and overall well-being. Jenny et al. [23] introduced the concept of proportionate care delivery based on available clinical data. This innovative approach streamlines care and ensures that resources are allocated efficiently, addressing patients’ needs more effectively [30]. By leveraging existing clinical data, healthcare providers can identify individuals at risk of high blood glucose levels, enabling proactive intervention and reducing the likelihood of complications associated with uncontrolled GDM.

Nonetheless, it is essential to acknowledge that challenges persist within GDM prediction. A common challenge encountered is the extensive array of variables associated with GDM [38]. The condition’s multifaceted nature means numerous factors must be considered, making selecting and weighing these variables complex [39]. While studies like that of Jie et al. (2022) have demonstrated the potential of different machine-learning models, addressing this variable complexity remains a significant challenge [26]. Researchers must continue refining their models and methodologies to accurately incorporate the full spectrum of relevant variables. Moreover, applying ensemble methods, such as stacking, underscores the aspiration to enhance predictive performance. While these methods promise to improve accuracy, they also introduce additional layers of complexity. Studies must balance model sophistication and practicality, ensuring that predictive models can be effectively implemented in real-world clinical settings.

As technology and healthcare data evolve, future research can leverage emerging opportunities. Integrating real-time data from wearable devices, exploring genetic data, and incorporating a more comprehensive range of health-related information are all promising avenues for improving predictive models. These advanced data sources have the potential to provide a more holistic understanding of GDM risk, leading to more accurate and timely predictions [40]. Furthermore, future research should consider the holistic context in which GDM occurs. Focusing on patient-centred outcomes and the social determinants of GDM can deepen our understanding of this condition. Factors such as access to healthcare, socioeconomic status, and lifestyle can significantly impact an individual’s risk of developing GDM. By considering these broader determinants, researchers and healthcare providers can develop more comprehensive and effective management strategies that address the medical aspects and the social and environmental factors influencing GDM.

## Limitations and strengths of review

This review explores various studies on predicting and detecting GDM through machine learning methods. It encompasses a wide range of study designs, population groups, and machine learning algorithms, providing an inclusive overview of this field’s current state of research. However, the studies included in this review span across different geographical regions and demographic profiles. While this diversity enriches the scope of the review, it can simultaneously limit the generalizability of findings. GDM risk factors and predictive models may exhibit variations among populations, and the review would benefit from a more thorough discussion of the implications arising from this variability. Additionally, this review primarily relies on studies published in English, which might introduce publication bias, potentially overlooking negative or inconclusive results less readily available in English literature.

## Conclusion

Predicting and early detecting GDM through machine learning techniques is a dynamic and evolving field. This review shows significant findings and trends across diverse studies, shedding light on the potential and challenges within this domain. The significance of early prediction in facilitating effective GDM management is striking, with numerous studies committed to crafting models capable of identifying GDM risk in the early stages of pregnancy. XGBoost emerged prominently as a consistent performer, showcasing superior predictive capabilities across various cohorts and time points. These models create opportunities for timely interventions and personalised care, ultimately improving outcomes for both mothers and infants. Nevertheless, the challenges at hand are notable. The vast array of variables associated with GDM poses a substantial hurdle in the quest for accurate prediction models. The selection and weighting of these variables remain intricate tasks, necessitating ongoing research and innovation in feature engineering. Furthermore, the emphasis on tailoring predictive models to specific populations, evident in studies focusing on Asian women or high-risk groups, underscores the importance of demographic-specific considerations. Predictive models must adapt to these groups’ unique characteristics and risk factors. The practicality of implementing proportionate care delivery based on readily available clinical data underscores the value of leveraging existing resources effectively. As technology and healthcare data continue to advance, there is an opportunity for future research to harness real-time data from wearable devices and genetic information to enhance predictive models further. These emerging data sources could revolutionise GDM prediction and early intervention. Focusing on patient-centred outcomes and exploring the role of social determinants in GDM prediction can deepen our understanding of this condition. It can pave the way for more comprehensive and effective management strategies considering medical variables and broader contexts in which GDM occurs. This review offers valuable insights and directions for future studies in GDM prediction through machine learning techniques.

## Data Availability

No new datasets were generated for this study. All data used are within this manuscript.

## Abbreviations

GDM: Gestational Diabetes Mellitus
T2DM: Type 2 Diabetes Mellitus
USPSTF: U.S. Preventive Services Task Force
SHAP: SHapley Additive exPlanations
TPOT: Tree-based Pipeline Optimization Tool
MeSH: Medical Subject Headings
MSE: Mean Squared Error
R2: R-squared (coefficient of determination)
MAE: Mean Absolute Error
BMI: Body Mass Index
OGTT: Oral Glucose Tolerance Test
LGBM: Light Gradient Boosting Machine
XGBoost: Extreme Gradient Boosting
SVM: Support Vector Machines
MLP: Multi-Layer Perceptron
KNN: K-Nearest Neighbors
LR: Logistic Regression
RF: Random Forest
ET: Extra Trees
BRF: Balanced Random Forest
GB: Gradient Boosting
ANN: Artificial Neural Network
HbA1c: Hemoglobin A1c
FPG: Fasting Plasma Glucose
TG: Triglyceride
TC: Total Cholesterol
HDL: High-Density Lipoprotein
SHAP: SHapley Additive exPlanations
TPOT: Tree-based Pipeline Optimization Tool
